# Surgeon Knowledge of the Pulmonary Arterial System and Surgical Plan Confidence Is Improved by Interactive Virtual 3D-CT Models of Lung Cancer Patient Anatomies

**DOI:** 10.3389/fsurg.2021.652428

**Published:** 2021-03-29

**Authors:** Kunal Bhakhri, Eoin R. Hyde, Sze M. Mak, Lorenz U. Berger, Sebastien Ourselin, Tom Routledge, Andrea Billè

**Affiliations:** ^1^Department of Thoracic Surgery, Guy's & St Thomas' Hospital, London, United Kingdom; ^2^School of Biomedical Engineering and Imaging Sciences, King's College London, London, United Kingdom; ^3^Department of Radiology, Guy's & St Thomas Hospital, London, United Kingdom

**Keywords:** survey, surgical planning, interactive virtual 3D model, computed tomography, robotic assisted thoracic surgery, minimal access surgery, lung cancer, 3D-CT

## Abstract

**Objective:** Interactive three-dimensional virtual models of pulmonary structures (3D-CT) may improve the safety and accuracy of robotic-assisted thoracic surgery (RATS). The aim of this study was to evaluate the impact of 3D-CT models as an imaging adjunct on surgical confidence and anatomical assessment for lobectomy planning.

**Methods:** We retrospectively analyzed the response of 10 specialist thoracic surgeons who each reviewed 10 pre-operative images of patients undergoing robotic-assisted lobectomy lung cancer cases from June to November 2018 in our institute, resulting in 100 data points. The number of arteries, veins, and bronchi entering the resected lobes were determined from the operation video recording by the operating surgeon. 3D-CT models were generated for each case and made available for online visualization and manipulation. Thoracic surgeons were invited to participate in the survey which consisted of evaluation of CT (control) and 3D-CT (intervention) models. A questionnaire regarding anatomical structures, surgical approach, and confidence was administered.

**Results:** Ten participants were recruited. 3D-CT models led to a significant (*p* < 0.003) increase in the surgeons' ability to correctly identifying pulmonary arteries entering the resection lobes in 35% (CT) and 57% (3D-CT) of cases. A significant (*p* < 1e-13) improvement in anatomy assessment and surgical plan confidence was observed for the 3D-CT arm, with median Likert scale scores of “2–Slightly easy” (CT) and “4–Very easy” (3D-CT).

**Conclusion:** The use of 3D-CT models for thoracic surgery planning increases the surgeon confidence in recognizing anatomical structures, largely by enhanced appreciation of anatomical variations in the segmental pulmonary arterial system. Further studies are needed to investigate if 3D-CT models can be used in providing precise information about segmental artery distribution and therefore surgical planning of sub-lobar resections.

**Graphical Abstract:**
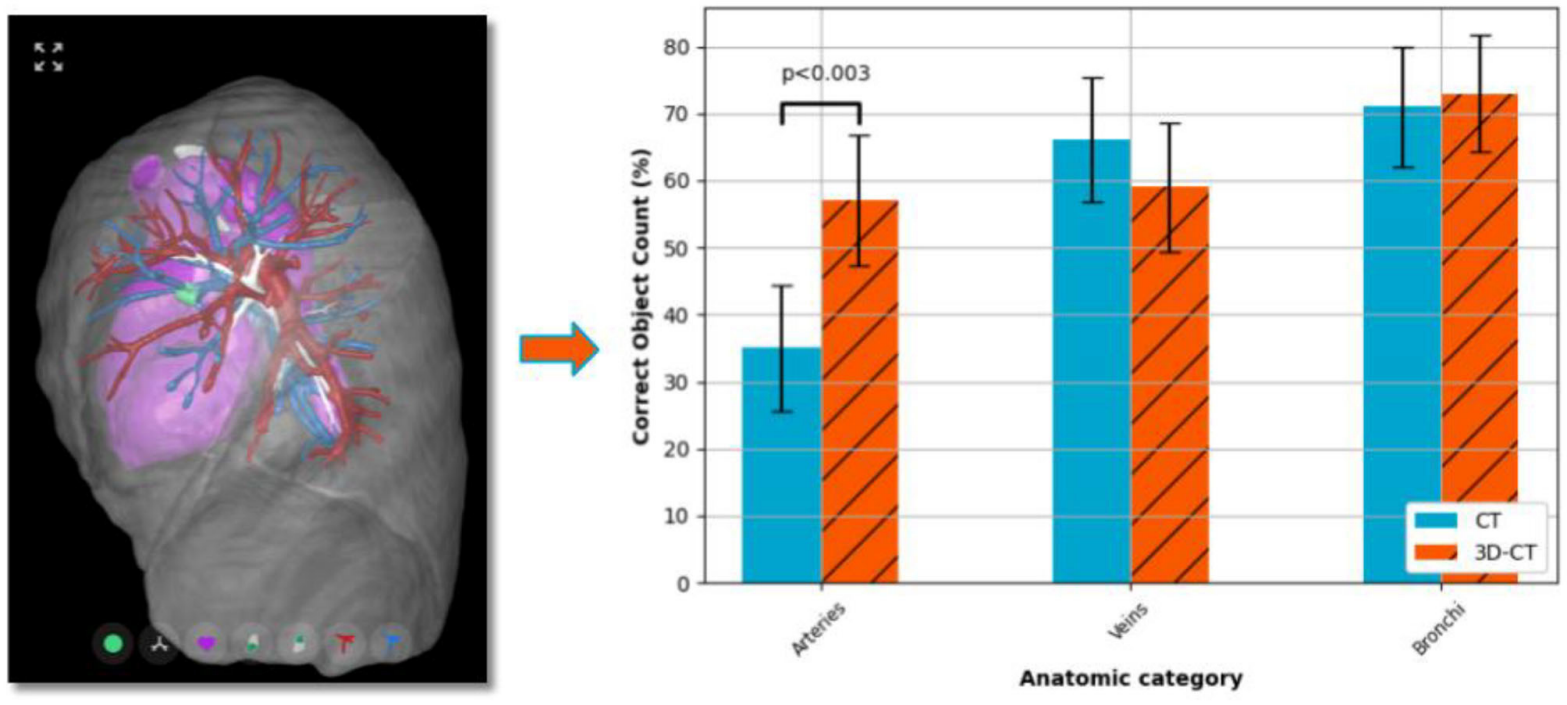
Interactive virtual 3D model of pulmonary anatomy enhances surgeon arterial knowledge.

## Highlights

- Patient-specific 3D-CT models improve the preoperative appreciation of the pulmonary arterial system and surgery plan compared to chest CT.
- Using a surgeon questionnaire, we successfully demonstrated that preoperative 3D-CT models can assist thoracic surgeons with surgical planning by allowing them to easily appreciate anatomical variation of critical pulmonary structures which may be more challenging to discern from CT data alone.

## Introduction

Video assisted thoracoscopic surgery (VATS) is the gold standard surgical approach for early stage lung cancer and, compared to thoracotomy, it provides benefits with regards to patient postoperative pain experience, recovery time, tissue trauma, and cosmetic results with equivalent oncologic outcome (1, 2). Similar to trends in other surgical areas, the proportion of cases being treated via robotic-assisted thoracic surgery (RATS) is on the rise, as some surgeons prefer RATS over VATS due to advantages in dexterity and degrees of freedom (3, 4). Doubtlessly, the use of robotics in thoracic surgery is still evolving in terms of its indications and applications, and the shift to RATS is not universal (5), perhaps due to RATS being associated with key challenges such as increased cost and a further reduction in tactile sensation when compared to VATS. However, as has already been noted (6), the high-powered console inherent in the robotic system lends itself to using pre-operative patient-specific data to assist in surgical education and practice (7) and intra-operative image guidance. The latter could improve surgical planning and it is generally hypothesized that an enhanced awareness of anatomical variation by the surgeon would in turn increase the precision of anatomic resection (8, 9). It is important to recognize segmental structures to reduce the risk of vascular and bronchial injuries due to inadvertent resection of additional segmental structures. This information can be difficult to capture from Computed Tomography (CT) alone. Standard-of-care multi-detector CT images, enhanced with contrast agent for better delineation of the pulmonary vasculature, was first combined with volume rendering software to provide a three-dimensional (3D) image of the patient's specific anatomy (9–11). More recently, modern medical image segmentation software has been applied to the contrast-enhanced CT scans to produce separable surface representations of the anatomical components (e.g., arterial, venous and bronchial systems). Our hypothesis is that interactive virtual 3D models build from CT angiography scans (3D-CT) allows for surgeons to obtain a clearer anatomical picture by use of differing colors and transparency amongst the components and deriving spatial measurements such as total lung volume (8, 12–14). To test this hypothesis, we invited a selection of UK-based thoracic surgeons, proficient in thoracic anatomy and surgical work up to participate in a survey assessing the anatomical understanding of specific cases from conventional CT images and the additional use of 3D-CT models.

## Methods

### Patient Selection and Imaging Data

**Figure 1:**
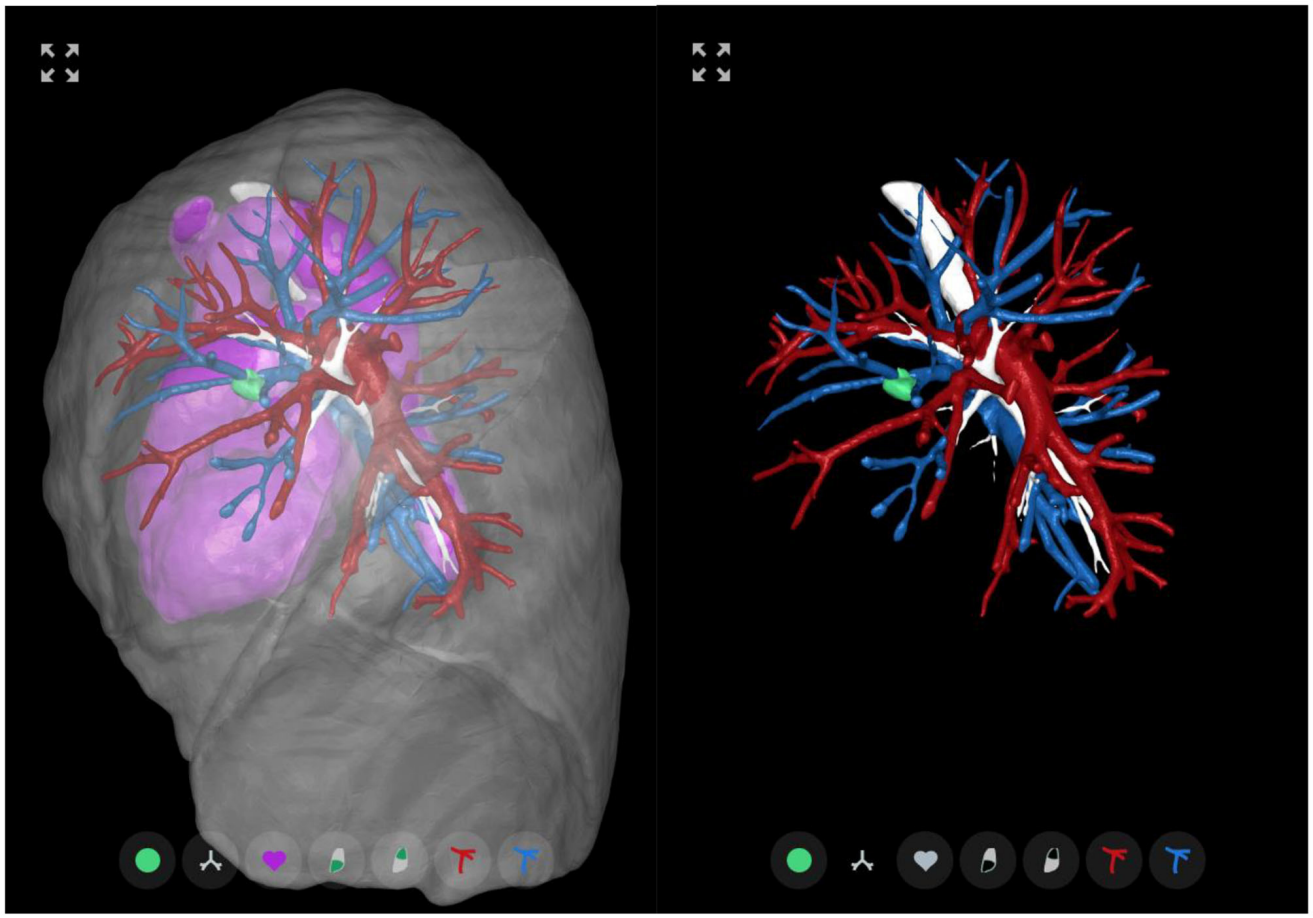
3D-CT example. Sample images representing the 3D-CT model available to the participants for the survey cases. 3D-CT model viewed along the sagittal axis from left-to-right of a patient case with a pulmonary left upper lobe nodule treated by robotic anatomical lung resection. (**Left**) Default object view with the airways, arteries, veins, heart and nodule shown at full opacity with the lobe surfaces shown in semi-transparency. (**Right**) To allow for a clearer view of the pulmonary vessels, the heart and lobes have been made fully transparent. In this case, one can see that the lingular artery runs anterior to the bronchus. The structure-to-colour keys are: tumor—green; bronchus—white; heart—pink; lobes—transparent gray; arteries—red; and veins—blue (see online version for color images).

Ten consecutive patients who had a robotic lung resection in 2018 were enrolled for this prospective evaluation. Those patients, as routine preoperative staging procedures, had chest and abdominal CT scans. All operations were recorded and the recordings were used to assess the number of vascular and bronchial structures to the lobe affected by the cancer. CT scans with minimum 1.25 mm slice thickness and contrast enhancement were acquired and patient-specific interactive virtual 3D models were made. The participants had to review first the CT and report the number of arterial branches, veins, and bronchi to be resected in order to complete the lobectomy. Afterwards, they reviewed the 3D model and similarly reported the numbers. This study design was intended to negate the potential confounding factor of having a different group of participants in each arm. Patient CT scans were anonymized and imported into a dedicated lung model generation pipeline within the medical imaging platform, *Innersight3D*™ (Innersight Labs Ltd., London, UK). This software was used to perform the image segmentation task, thereby partitioning the scan voxels into pre-selected classes, namely, background, pulmonary artery, pulmonary vein, normal lung parenchyma, and abnormal lung tissue (study models can be viewed from following the publications page links provided at https://innersightlabs.com). Image processing was performed by a research scientist with 9 years of medical image processing experience with the guiding assistance of a consultant radiologist specializing in thoracic oncology to ensure that 3D reconstruction of pulmonary vessels exactly corresponded to CT findings. The output of the software package was a set of Visualization ToolKit (VTK) files detailing the surfaces of all objects of interest for each case where each virtual 3D model could be interacted with including viewing from any angle, and selective structure visibility and transparency (Figure 1). This study was approved by the local institutional review board at Guys and St Thomas' NHS Foundation Trust, London.

### Surgical Approach

All patients had a robotic anatomical lung resection using the *da Vinci*^®^ *Xi* Surgical System (Intuitive Surgical Inc., Sunnyvale, CA 94086, USA). The surgical approach consisted of four robotic ports (two 8 mm and two 12 mm) and one utility port of 15 mm for the bedside assistant. Dissection was performed with fenestrated bipolar forceps and spatula. Full lymphadenectomy was performed and the fissure was approached and opened in every case. The vein(s) for the target lobe(s) were identified and stapled with a robotic vascular staple. Arterial lobar branches were identified, isolated and stapled separately. Target lobar bronchus was identified and stapled. The fissure was completed with diathermy and robotic staplers to minimize postoperative air leak. The number of vascular and bronchial structures were carefully documented on the operative notes and the recording of the procedure was used to count the numbers of vascular and bronchial structures. Any anatomical variations or aberrant structures was also recorded.

### Survey Approach

Survey participants were restricted to thoracic surgeons who were thoracic surgery board certified, none of the participants were involved in the pre and postoperative care of the patients involved in the study. The survey was hosted on a bespoke website managed by Innersight Labs Ltd. to provide secure and authorized access to the anonymized CT scans and 3D-CT models. The survey consisted of a standard CT scan and a 3D reconstruction for each patient. The participants had to review first the CT and report the number of arterial branches, veins, and bronchi to be resected in order to complete the lobectomy. Afterwards, they reviewed the 3D model and similarly reported the numbers of the structures. Each questionnaire was the same for each case and study arm. The questionnaire was designed to investigate surgeon knowledge of the patient anatomy (Table 1). The questionnaire also asked participants to note specific anatomical abnormalities if encountered and to score the ease of use of the 3D-CT model based on their survey experience.

**Table 1:** Questionnaire.

**Question**	**Response options**
Please select the number of arterial branches entering the lobe to be resected	1–10
Please select the number of veins entering into the lobe to be resected	1–5
Please select the number of bronchi entering into the lobe to be resected	1–5
How easy was it for you to assess the patient anatomy and produce an operational plan that you had confidence in? Please choose one of: 1–Not at all easy, 2–Slightly easy, 3–Easy, 4–Very easy, 5–Extremely easy.	1–5

*Questionnaire with regards to the surgeon participant's appreciation of anatomical structure and surgical planning confidence under control (CT only) and intervention (3D-CT) surgery planning methods*.

### Statistical Analysis

No sample size calculation was performed. Categorical variables were reported as frequencies (percentages) or median values with interquartile range (IQR). Categorical variables were compared using a Fisher exact test where the participant value for the object number was converted to a Boolean correct/incorrect relative to the ground truth value. Qualitative scores (i.e., Likert items) are quantified by the nonparametric Wilcoxon signed-rank test with Pratt treatment of zero-differences. For ease of anatomical assessment, the null hypothesis tested is that the case-matched distribution of the intervention score minus the control score is symmetric about zero, i.e., that there is no difference between study arms. A two-sided *p*-value < 0.05 was considered statistically significant. Statistical analysis was performed using Python's statistical functions (Python 3.6.9; SciPy v1.5.0).

## Results

### Patient and Participant Demographics

Ten patients underwent robotic lobectomy with a median age of 68 (interquartile range 59–73). There were 7 females (70%) and 3 males (30%). Their combined average (±SD) body mass index (BMI) was 27.2 ± 5.6. There were two current smokers (20%), seven former smokers (70%) and one patient never smoked (10%). Other patient characteristics were summarized in Table 2. Seven patients had left anatomical lung resection, three of which were upper and four were lower lobectomies. Three patients had a right lung resection: one bilobectomy (middle and lower lobe), one lower and one upper lobectomy. For each case, the number of arteries, veins, and bronchi entering the lobes to be resected are listed in Table 3.

**Table 2:** Preoperative baseline characteristics and comorbidities.

	**RATS (*n* = 10) *n* (%)**
Age, median (IQR)	68 (59, 73)
Sex	
Female	7 (70)
Male	3 (30)
BMI, mean ± SD	27.2 ± 5.6
Smoking history	
Current, *n* (%) - avg pack/year	2 (20) - 25
Former, *n* (%) - avg pack/year	7 (70) - 31
Never, *n* (%)	1 (10)
ECOG performance status	
Score 0	3 (30)
Score 1	5 (50)
Score 2	2 (20)
Diabetes mellitus	2 (20)
COPD	3 (30%)
FEV1 (L), mean ± SD	2.4 ± 0.7
FVC (L), mean ± SD	3.5 ± 1.3

*IQR, interquartile range; BMI, body mass index; COPD, chronic obstructive pulmonary disease; FEV1, forced expiratory volume in 1 second; FVC, forced vital capacity; RATS, robotic-assisted thoracic surgery; ECOG, Eastern Cooperative Oncology Group*.

**Table 3:** Case anatomic variability.

**Case #**	**Number of arteries**	**Number of veins**	**Number of bronchi**	**Resection target lobe(s)**
1	4	1	1	LUL
2	2	1	1	LLL
3	3	1	1	LUL
4	4	1	1	LUL
5	2	1	1	LLL
6	2	1	1	LLL
7	2	1	1	RLL
8	3	2	1	RUL
9	3	2	2	RLL/RML
10	4	1	1	LUL

*Participants were asked to identify the number of arteries, veins, and bronchi entering lobes to be resected for 10 retrospective lung cancer cases. Two methods were available to help determine the numbers, using standard Computed Tomography (CT), and when given access to an additional interactive virtual 3D model of the target lung (see Figure 1). Resection target lobes are identified by left (L) or right (R) lungs in the first letter, and upper (U), middle (M), or lower (L) lobes in the second letter*.

Ten thoracic surgeons were identified to complete the survey The median number of lung resections conducted per participant per annum was in the range 75–120

### Anatomic Variation

**Figure 2:**
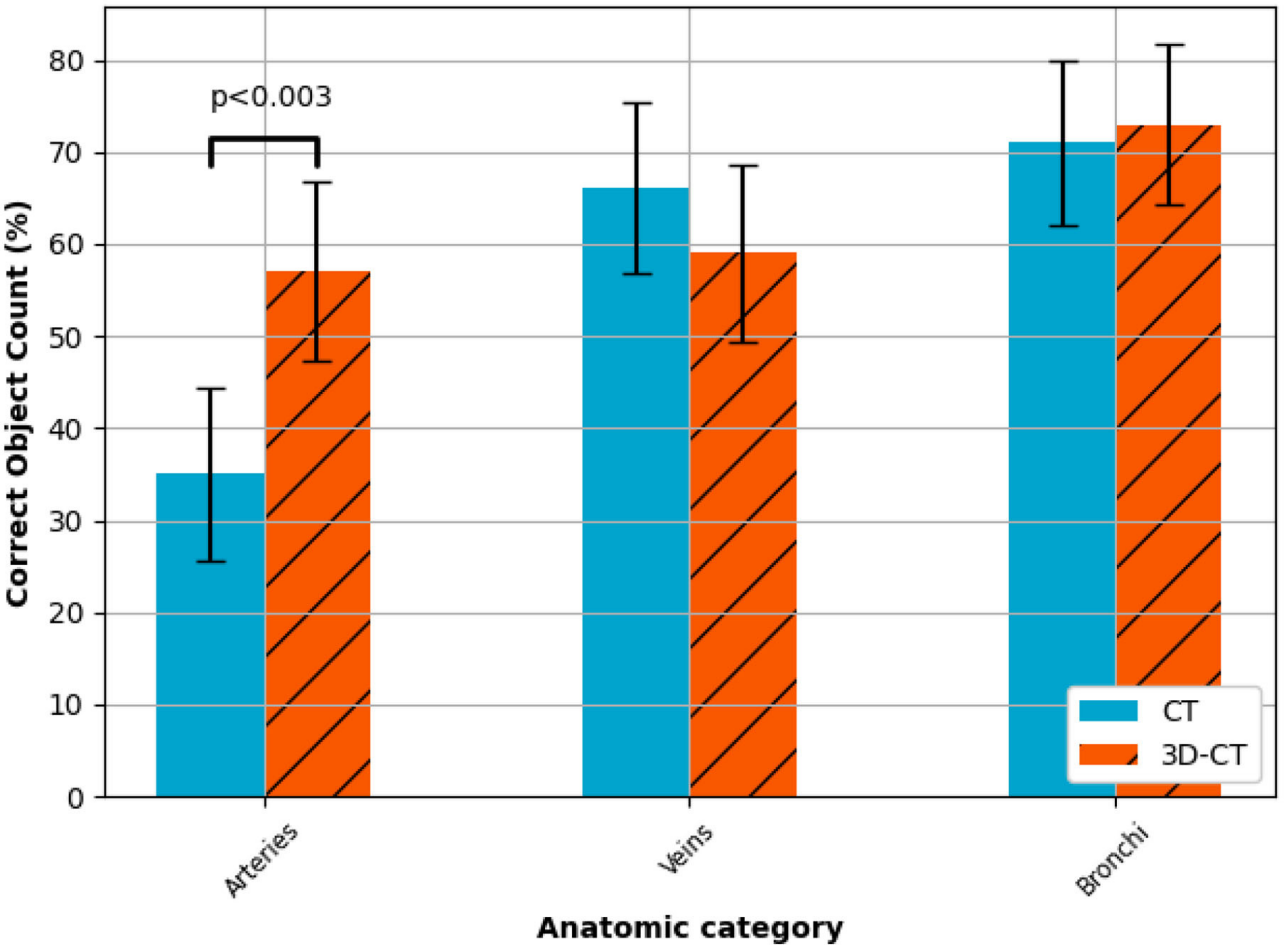
Anatomical understanding. Participant results from the anatomical understanding questions (survey questions 1–3), i.e., count the arteries, veins and bronchi entering the lobes to be resected. A statistically significant (*p* < 0.05) improvement in the correct identification of arterial vessels was observed for the 3D-CT surgery planning method. There was no significant difference between the planning methods for identifying the number of either the veins or the bronchi.

Comparing the accuracy of identifying vascular structures, the percentage of correct numbers of arteries entering the target lobe(s) across all cases was significantly larger for the 3D-CT arm (57% of responses correct) when compared to the CT only arm (35% of responses correct) (*p* < 0.003). There was no significant difference between the arms for either the veins or the bronchi structures (Figure 2).

### Surgeon Opinion on Ease of Assessment

**Figure 3:**
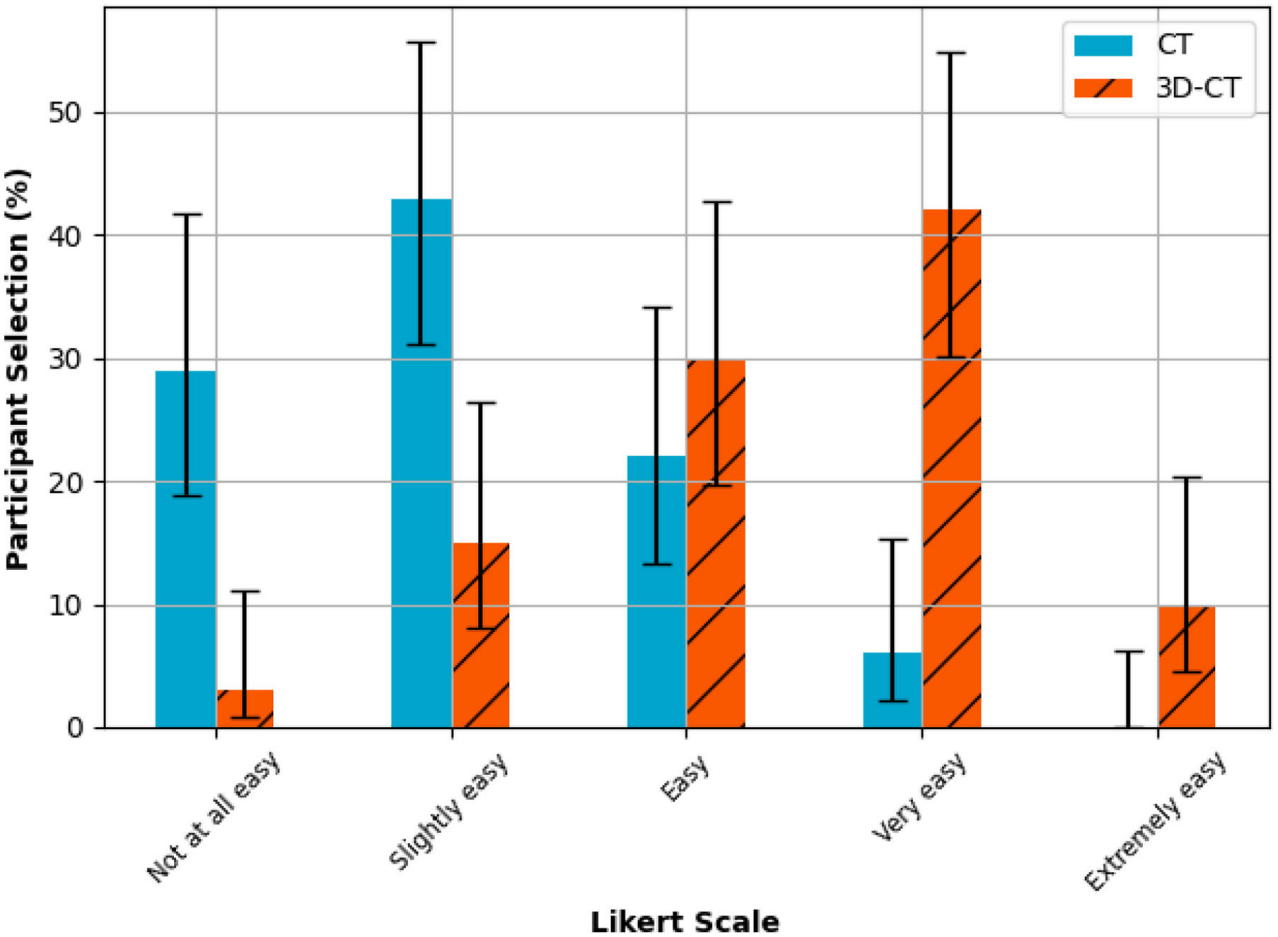
Ease of anatomy assessment. Bar chart (with 95% confidence interval) for the percentage response by surgeons to the question of how easy it was to produce a surgery plan for each case that they could have confidence in for each of the two surgery planning methods on a Likert scale. The median scores are “2–Slightly easy” for the CT arm and “4–Very easy” for the 3D-CT arm.

**Figure 4:**
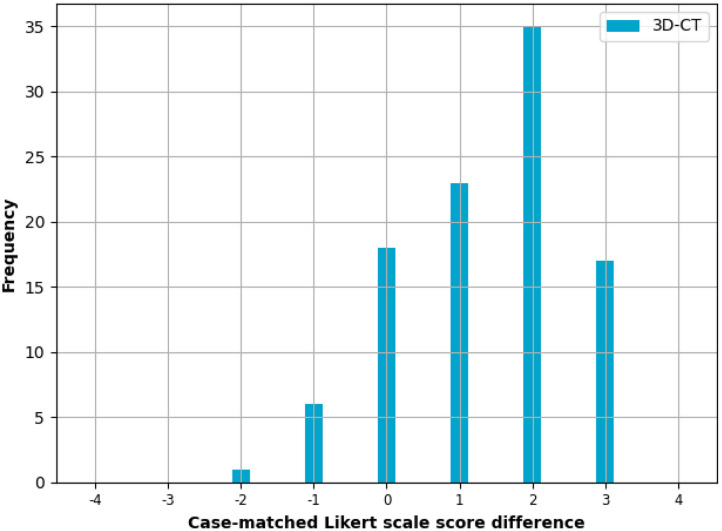
Case-matched assessment. Case-matched difference in the Likert scale score for surgeons' ease of surgical planning between the 3D-CT and CT only planning methods. A positive value means the surgeon preferred using the 3D-CT method, a value of zero means the surgeons found no difference between the methods, and a negative value means the surgeon preferred the CT only method. If the two methods are equal, one would expect a symmetric distribution centred on zero. A statistically significant (*p* < 0.05) improvement in surgeon opinion on ease and confidence in planning was observed, with a median difference of 2 when the CT Likert scale score was subtracted from the 3D-CT Likert scale score.

Comparing the ease of anatomy assessment averaged across all cases, the median Likert scale scores were “2-Slightly easy” for the CT arm and “4-Very easy” for the 3D-CT arm (Figure 3). Comparing the case-matched distribution of the CT only Likert scale score minus the 3D-CT arm Likert scale score, the median value was 2, with 7 (7%) negative scores (CT better) and 75 (75%) positive scores (3D-CT better). The remaining 18 (18%) scores had a neutral value of zero signifying no perceived difference in anatomical assessment ease for those cases. A statistically significant (*p* < 1e-13) improvement in surgeon opinion on ease and confidence in planning was observed (Figure 4).

## Conclusions

In the era of minimally invasive surgery and with the increasing prevalence of segmentectomy due to early stage lung cancer, careful tumor localization and identification of vascular structures is essential for anatomical lung resections. Arterial anatomy is variable in up to 20% of cases and while venous and bronchial structures are more standard (15), anomalies are still possible and thus preoperative identification is essential. In this study, we have shown that the use of 3D-CT models, alongside routinely acquired CT scans, can improve surgeon knowledge of the patient anatomy of the pulmonary arterial system relevant to anatomical lung resections by 22% on average (Figure 2), aiding the surgeon in achieving the best surgical outcomes with limited resection. It is possible that a similar improvement in surgeon knowledge would replicate across the pulmonary venous system, to show this would require a larger dataset. In this study, only 2/10 cases had two veins to be dissected with the remaining cases having a single vein. The surgeons undertaking the survey assess the bronchial anatomy preoperatively with fiber-optic bronchoscopy, but accessory bronchi may be present and better understanding of airways anatomy can improve surgical planning.

With screening lung cancer programs, the number of early stage lung cancer detected is increasing. In those patients sub-lobar resection may represent the best treatment option. Segmentectomy is a valid surgical option for a nodule < 2 cm which requires a more challenging dissection within segmental planes. We have presented three-dimensional reconstructions that could assist in the important task of nodule localization in order to guarantee good surgical margins, helping to plan the segmentectomy in real cases, in particular on how to complete the inter-segmental fissure. Various computational techniques exist on how to estimate the inter-segmental fissure when it cannot be directly observed in the CT scan (13, 16). Virtual 3D models with segmental surfaces derived from medical scans and statistical priors can complement existing approaches to fissure identification such as bronchial ligation (17), arterial ligation (18), and indocyanine green (ICG) (19). In future work, we plan to add this additional information to the virtual 3D models.

Through this retrospective study, we have provided further evidence that surgeons can understand patient anatomy better from using similar 3D reconstructions alongside standard CT, and increased confidence from gaining this knowledge (Figure 3) could lead to better surgical planning and aid in precision sub-lobar resection, as has been speculated previously by Xue et al. (20). There is a paradigm shift in thoracic surgical oncology to ensure lung cancer survival by carrying out early screening. Further a better understanding of the cancer genetics in particular adenocarcinoma spectrum will result in the tumor board offering surgery to patients with multiple ground glass nodules. 3D modelling surgical planning tools will be increasingly used to map and plan surgical strategy (21).

### Study Limitations

There are some limitations with regards to this study. Fully completing the questionnaire was a lengthy commitment as it involved answering the same set of four questions twenty times (10 cases × 2 study arms). Extrapolation to the general minimal access thoracic surgeon should be treated with caution.

Assessment of inter-rater agreement is not applicable to this study due to the unbalanced results dataset as the majority of surgeons found the anatomical assessment easier when using the 3D-CT model (Figure 3).

This preliminary study does not account for all anatomical challenges that a surgeon may encounter intra-operatively. For example, the authors did not attempt to detect, grade, or display mediastinal lymph nodes which could provide useful information to surgeons during the pre-operative planning stage to assist with lymph node dissection and reduce intraoperative complications (22). This decision was due to a shortage of annotated data sets to use for lymph node detection and a sensitivity (also known as the true positive rate or recall) in the region of 85% which was considered not reliable enough for clinical deployment (23). Neither CT or 3D-CT models incorporate the effects of intra-operative organ movement, for example caused by lung deflation, which can lead to spatial disorientation of the surgeon and/or induce landmark deviations from pre-operative data (16, 24).

While there were more questions that could have been posed to the participants, for example the position and direction of key vascular structures, the authors had to keep in mind the time demands made on high-volume expert surgeons. Fully completing the questionnaire was a lengthy commitment as it involved answering the same set of four questions twenty times (10 cases × 2 study arms).

Finally, this study did not investigate clinical outcomes. While our results clearly indicate that surgeons are absorbing more correct anatomical information via the 3D-CT models, it does not prove that this additional information will necessarily lead to improved patient outcome. A prospective randomized controlled trial (RCT) examining primary outcomes such as length of stay, complication incidents, blood loss, and operative time will be required.

## Data Availability Statement

The original contributions presented in the study are included in the article/supplementary material, further inquiries can be directed to the corresponding author/s.

## Author Contributions

KB and EH: data collection, formulation interpretation, and write up. SM: expert input from a radiological perspective. LB and SO: data interpretation of 3D software. TR: involved with utilization of technology and expert surgical input into its utilization. AB: principal investigator also involved with utilization of technology and expert surgical input into its utilization. All authors contributed to the article and approved the submitted version.

## Conflict of Interest

The authors declare that the research was conducted in the absence of any commercial or financial relationships that could be construed as a potential conflict of interest.

## Abbreviations

BMI: Body mass index
CT: Computed tomography
ICG: Indocyanine green
IQR: Interquartile range
RATS: Robotic-assisted thoracoscopic surgery
RCT: Randomized controlled trial
VATS: Video assisted thoracoscopic surgery
2D: Two-dimensional
3D: Three-dimensional.
